# Anaerobic digestion of soybean molasses: comparison between single and two-stage systems, operational temperature and microbial communities

**DOI:** 10.3389/fbioe.2026.1936766

**Published:** 2026-09-15

**Authors:** Isabela Mehi Gaspari Augusto, Ariane Moreira Ozú, Pedro Bissolotti Vendrasco, Lia Paula Poloni Batista, Armando Zanone, Roberta Albanez, Giovanna Lovato, Suzana Maria Ratusznei, José Alberto Domingues Rodrigues

**Affiliations:** 1 Department of Chemical Engineering, Mauá School of Engineering, Mauá Institute of Technology (EEM/IMT), São Caetano do Sul, São Paulo, Brazil; 2 Department of Environmental Engineering Sciences, São Carlos School of Engineering, University of São Paulo, São Carlos, São Paulo, Brazil; 3 Department of Chemical Engineering, Institute of Chemistry, São Paulo State University, Araraquara, São Paulo, Brazil

**Keywords:** anaerobic digestion, AnSBBR, biohydrogen, methane, microbial community, organic loading rate, soybean molasses, two-stage process

## Abstract

Soybean molasses is an abundant, low-value by-product of soy protein concentrate production whose valorization through anaerobic digestion remains little explored, and no two-stage system has yet been reported for this substrate. This study evaluated methane production in a methanogenic anaerobic sequencing batch biofilm reactor fed with the effluent of a thermophilic acidogenic reactor treating soybean molasses, assessing the influence of applied organic loading rate (4–12 kg-COD/m^3^/d) and operational temperature (30 or 55 °C) on performance, stability, and microbial community structure. An energy analysis then compared four single- and two-stage configurations. The reactor was stable at every loading rate, consuming the volatile acids of the acidified influent almost completely while generating alkalinity internally. Mesophilic operation outperformed thermophilic operation, reaching a methane productivity of 152.8 mol-CH_4_/m^3^/d and a yield of 13.1 mol-CH_4_/kg-COD at the highest load, 69% and 62% above the thermophilic values. Community analysis showed that temperature governed structure: mesophilic reactors were diverse and acetoclastic, whereas thermophilic reactors were dominated by specialists relying on syntrophic acetate oxidation. Splitting the process into two stages did not increase the overall energy yield relative to single-stage methanogenesis, which was already efficient; its value lies instead in hydrogen co-production and process stability, with a thermophilic-acidogenic and mesophilic-methanogenic pairing representing the most rational and energetically competitive configuration for this by-product.

## 1 Introduction

The intensifying conflict between economic growth and environmental sustainability, driven by fossil fuel dependence and greenhouse gas emissions, has renewed interest in recovering energy from industrial by-products within a circular bioeconomy framework (Ghisellini et al., 2016; Mancini and Raggi, 2021). Anaerobic digestion (AD) is particularly well suited to this purpose, converting organic wastes into biofuels through a process with a more favorable energy balance and lower capital requirements than gasification or pyrolysis, while yielding a digestate that can be valorized as fertilizer (Gunes et al., 2019).

Soybean molasses, a low-value brownish viscous by-product, is generated when de-oiled soybean meal is washed with an alcohol–water mixture during soy protein concentrate processing, exiting the evaporation step at approximately 50 °C (de Mello et al., 2021). Roughly 232 kg of molasses are produced per ton of soybean meal (Siqueira et al., 2008), and Brazil alone generates on the order of millions of tons annually, with single large biorefineries producing 300–515 tons per day (Augusto et al., 2024). Lacking a consolidated market and presenting disposal difficulties, this stream constitutes an environmental liability that AD could convert into an energy asset. Its composition is, however, distinctive: although rich in carbohydrates, a substantial fraction occurs as the oligosaccharides raffinose and stachyose, whose higher polymerization degree and requirement for α-galactosidase hydrolysis make them more recalcitrant than the simple sugars dominating sugarcane or beet molasses (Augusto et al., 2024).

Among AD configurations, the Anaerobic Sequencing Batch Biofilm Reactor (AnSBBR) offers tolerance to high organic loading rates, flexible operation through adjustable feeding mode and cycle length, and high biomass retention via immobilization - advantages that are especially valuable for agro-industrial by-products subject to seasonal availability and concentration fluctuations (Lovato et al., 2015; Paulinetti et al., 2022). Two parameters consistently determine the success or failure of such reactors: the applied organic loading rate (OLR_A_), which can boost methane output but also trigger acidification and overload, and the operational temperature, which governs efficiency, stability, and economic viability (Nie et al., 2021; Zhang et al., 2022).

Despite its potential, soybean molasses remains scarcely explored as an AD substrate, and the prior work that exists points to an unresolved set of trade-offs. For methane, a mesophilic AnSBBR achieved strong performance (140.9 mol-CH_4_/m^3^/d, 11.0 mol-CH_4_/kg-COD) and proved robust at high load (Paulinetti et al., 2023), whereas a thermophilic AnSBBR yielded marginally higher methane at moderate loads but destabilized at 20 kg-COD/m^3^/d, revealing a clear performance-versus-stability trade-off rooted in the reduced microbial diversity selected at 55 °C (Paulinetti et al., 2024). For hydrogen, mesophilic dark fermentation performed poorly, with homoacetogenesis consuming up to 59.9% of the hydrogen produced and endogenous substrate microorganisms displacing hydrogen producers (Augusto et al., 2024). Raising the temperature to 50 °C resolved much of this limitation, increasing productivity to 51.4 mol-H_2_/m^3^/d by suppressing hydrogen-consuming pathways and selecting *Thermoanaerobacterium*, *Clostridium*, and *Ethanoligenens*, while 55 °C and 60 °C degraded performance through excessive loss of diversity (Augusto et al., 2025). Notably, 50 °C also coincides with the temperature at which molasses leaves the soy protein concentrate process, favoring thermophilic acidogenesis on energetic grounds.

These findings collectively motivate a two-stage approach, in which acidogenesis and methanogenesis are physically separated. Phase separation allows each stage to operate near its own optimum, lets the acidogenic reactor act as a metabolic buffer that removes inhibitory components and dampens pH fluctuations before they reach the methanogens, and enables the recovery of both hydrogen and methane (Lovato et al., 2020; Cremonez et al., 2021). Two-stage AD has substantially enhanced energy recovery from complex or oligosaccharide-rich substrates, including a 3.3-fold improvement for lignocellulosic *Agave tequilana* hydrolysates (Arreola-Vargas et al., 2016). Critically, the choice of temperature for each stage is not arbitrary: for sugarcane vinasse, a thermophilic acidogenic reactor paired with a mesophilic methanogenic reactor delivered the highest energetic yield under high-load, low-biodegradability conditions, owing to the greater functional redundancy and shock resistance of mesophilic methanogenic communities (Ramos et al., 2021). This pairing also aligns with industrial practice, since the feed enters hot while the digestate must be cooled for downstream use.

Despite this convergent rationale, no two-stage AD system has yet been reported for soybean molasses. Building on the thermophilic dark fermentation optimum established for this substrate, the present study evaluates methane production from the effluent of a thermophilic acidogenic AnSBBR (Augusto et al., 2025) in a second-stage methanogenic AnSBBR, investigating the influence of applied organic loading rate (4–12 kg-COD/m^3^/d) and operational temperature (30 or 55 °C) on performance and stability. An energy analysis was subsequently performed to identify which configuration (single-stage or two-stage, thermophilic or mesophilic) achieves the highest energy yield, and the microbial communities of all systems were compared to link community structure to process behavior.

## 2 Materials and methods

The experimental data analyzed in this work were generated in a methanogenic AnSBBR fed with the effluent of a thermophilic acidogenic AnSBBR treating soybean molasses. To enable the energy analysis and the comparison between single- and two-stage configurations, previously published data from three systems treating the same soybean molasses were also used: the thermophilic acidogenic (hydrogen-producing) reactor (Augusto et al., 2025); the mesophilic single-stage methanogenic reactor (Paulinetti et al., 2023); and the thermophilic single-stage methanogenic reactor (Paulinetti et al., 2024).

### 2.1 Anaerobic sequencing batch biofilm reactor

Figure 1 shows a scheme of the system used for methane production from the effluent of a thermophilic acidogenic AnSBBR that had treated soybean molasses for hydrogen production.

**FIGURE 1:**
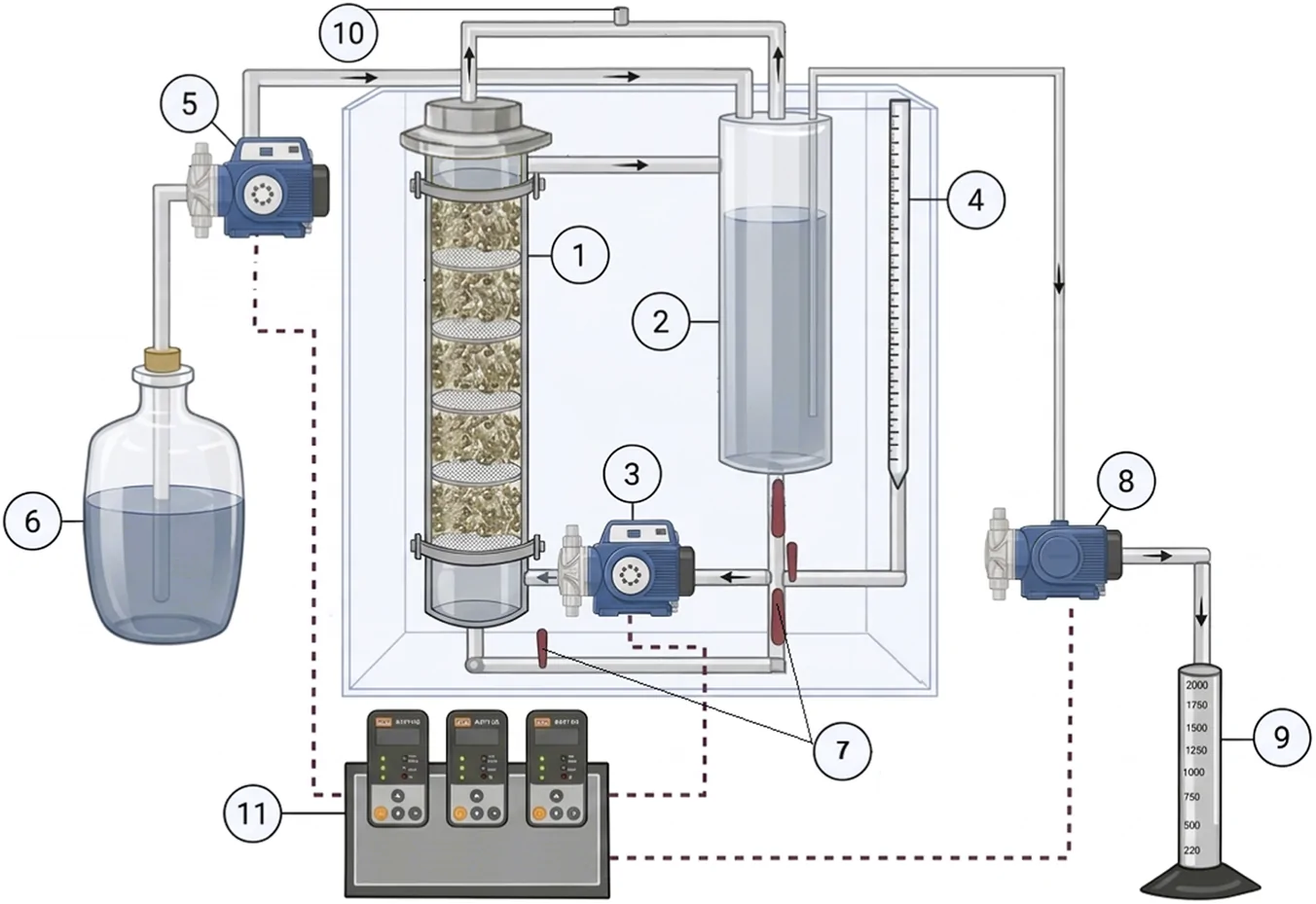
Scheme of the AnSBBR with recirculation. [Notation: 1 – Reactor with immobilized biomass; 2 – Side reservoir; 3 – Recycle pump; 4 – Flow meter; 5 – Feed pump; 6 – Wastewater reservoir; 7 – Discharge valves for purging; 8 – Discharge pump; 9 – Effluent outlet; 10 – Biogas outlet; 11 – Automation system].

The reactor consisted of a cylindrical acrylic column 540 mm in height, with a 100 mm external diameter and a 3.5 mm wall thickness, providing a total volume of 4.0 L. It held 1.6 L of liquid medium together with the inert support and immobilized biomass (2.0 L). The inert support containing the immobilized biomass was held between perforated stainless-steel plates that divided the column into five sections to prevent bed compaction. A 20 mm compartment at the base of the reactor promoted uniform wastewater distribution and prevented the formation of preferential flow paths, while a 40 mm headspace at the top served as a biogas collector (CH_4_ and CO_2_).

The liquid phase was recirculated through a side reservoir consisting of a cylindrical acrylic vessel 300 mm in height, with a 100 mm external diameter and a 3.5 mm wall thickness (total capacity of 2.1 L), which held 1.0 L of liquid medium. Recirculation was driven by a Prominent® diaphragm pump (model Beta/5, maximum capacity 30 L/h), and a 100 mL graduated cylinder was coupled to the recirculation line for flow-rate measurement. The total volume of liquid medium in the system, that is, the reactor’s working volume, was therefore 2.6 L.

Feeding and discharge were performed with Prominent® diaphragm pumps, models Beta/4 (maximum capacity 23 L/h) and Beta/5 (maximum capacity 30 L/h), respectively, each controlled by a Coel® automatic timer (model RTST/20) that programmed the feed, reaction, and discharge stages of the sequencing batch operation. The reactor was housed in a temperature-controlled chamber maintained at 30 °C ± 1 °C or 55 °C ± 1 °C.

The inoculum for mesophilic operation was obtained from an up-flow anaerobic sludge blanket (UASB) reactor treating poultry slaughterhouse wastewater, whereas the inoculum for thermophilic operation was obtained from a UASB reactor treating sugarcane stillage wastewater. The corresponding total solids (TS) concentrations were 62.0 and 26.9 g/L, and total volatile solids (TVS) concentrations were 51.0 and 16.5 g/L, respectively. Biomass was immobilized on the inert support according to Zaiat et al. (1994). The support consisted of 1 cm polyurethane foam cubes with an apparent density of 23 kg/m^3^ and a porosity of 95%.

The influent was the effluent of the thermophilic acidogenic reactor, rich in typical fermentative metabolites, namely, volatile acids (acetic, propionic, butyric, and lactic acids) and ethanol. Because this effluent was acidic (pH ≈ 4.0–5.0), its alkalinity was supplemented with sodium hydroxide (NaOH) and sodium bicarbonate (NaHCO_3_) to raise the influent pH to 7.0–7.5 before it was fed to the methanogenic reactor.

Influent and effluent samples were monitored according to the Standard Methods for the Examination of Water and Wastewater (APHA/AWWA/WEF, 2012). Alkalinity was determined according to Ripley et al. (1986) and total carbohydrates according to Dubois et al. (1956). The intermediate compounds of anaerobic metabolism (acetic, propionic, and butyric acids, and ethanol) were quantified with an Agilent Technologies® 7890 gas chromatograph equipped with a flame ionization detector (FID), automatic head-space injection (GC Sampler 80), and an HP-Innowax column. Biogas composition was analyzed with the same Agilent Technologies® 7890 gas chromatograph equipped with a thermal conductivity detector (TCD) and a GS-Carbonplot column, and the volumetric biogas production was measured with a Ritter Milligascounter gas meter.

### 2.2 Stability and performance indicators

The reactor’s stability and performance were assessed using the following indicators, calculated according to Lovato et al. (2016): organic matter removal efficiency, based on chemical oxygen demand (COD), for total (unfiltered, ε_T_) and soluble (filtered, ε_S_) samples (%); applied (OLR_A_) and removed (OLR_R_) organic loading rates (kg-COD/m^3^/d); methane molar productivity (MPr, mol-CH_4_/m^3^/d) and methane molar yield per applied (Y_A_) and per removed (Y_R_) organic load (mol-CH_4_/kg-COD).

### 2.3 Experimental procedure

The AnSBBR with recirculation of the liquid phase was operated under six experimental conditions, as shown in Table 1. Throughout the entire experimental procedure, the cycle length was fixed at 6 h and the feed time at 3 h, characterizing a fed-batch operation. Each condition was maintained until stability was reached, defined as a standard deviation below 10% for all monitored variables, and was then sustained for a minimum of 10 additional days, corresponding to at least 40 sequential batch cycles under stable operating conditions.

**TABLE 1:** Experimental conditions.

Range	Condition	C_INF_ (mg-COD/L)	OLR_A_ (kg-COD/m^3^/d)	Duration (days)	T (°C)
Mesophilic	1	2,600	4.0	34	30
	2	5,200	8.0	22	
	3	7,800	12.0	21	
Thermophilic	4	2,600	4.0	37	55
	5	5,200	8.0	28	
	6	7,800	12.0	25	

Notation: C_INF_, influent concentration; OLR_A_, applied organic loading rate; T, Operational temperature.

Conditions 1–3 investigated the influence of the applied organic loading rate under mesophilic conditions (30 °C), and conditions 4–6 investigated the same effect under thermophilic conditions (55 °C).

Each 6 h cycle began with the feeding of 1.0 L of influent over 180 min (50% of the cycle length), followed by a reaction phase under continuous recirculation of the liquid phase. At the end of the cycle, recirculation was interrupted and 1.0 L of effluent was discharged over 10 min, leaving a residual volume of 1.6 L retained in the reactor. A 1 min interval was then allowed to synchronize the timer-controlled feed and discharge pumps before the next cycle began, so that four cycles were completed per day. Minor variations in the volumes fed and discharged by the pumps occurred during operation; these account for the difference between the nominal OLR_A_ (Table 1) and the experimental OLR_A_ (Table 2).

**TABLE 2:** Monitored parameters and performance indicators for all conditions.

Parameter	Mesophilic			Thermophilic		
	Condition 1	Condition 2	Condition 3	Condition 4	Condition 5	Condition 6
C_INF_ (mg-COD/L)	2,629 ± 74^(24)^	5,251 ± 45^(14)^	7,781 ± 138^(14)^	2,633 ± 92^(24)^	5,229 ± 104^(18)^	7,621 ± 401^(12)^
ε_ST_ (%)	91 ± 2^(20)^	94 ± 2^(14)^	95 ± 1^(14)^	87 ± 3^(16)^	89 ± 1^(17)^	91 ± 2^(17)^
ε_SF_ (%)	93 ± 2^(20)^	95 ± 2^(14)^	96 ± 1^(14)^	90 ± 1^(16)^	91 ± 1^(17)^	92 ± 1^(17)^
C_Carb-INF_ (mg-Carb/L)	875 ± 27^(22)^	1,510 ± 46^(13)^	2,235 ± 119^(14)^	878 ± 31^(24)^	1744 ± 36^(18)^	2,177 ± 114^(17)^
ε_CT_ (%)	97 ± 1.2^(23)^	98 ± 0.8^(14)^	98 ± 0.1^(14)^	97 ± 1.1^(17)^	97 ± 0.4^(17)^	97 ± 0.9^(17)^
ε_CF_ (%)	98 ± 0.7^(23)^	99 ± 0.4^(14)^	99 ± 0.2^(14)^	98 ± 0.7^(16)^	98 ± 0.2^(17)^	98 ± 0.5^(17)^
pH_INF_	7.5 ± 0.3^(13)^	7.7 ± 0.2^(11)^	7.7 ± 0.3^(13)^	7.5 ± 0.3^(17)^	7.5 ± 0.2^(15)^	7.6 ± 0.2^(13)^
pH_EFF_	7.5 ± 0.1^(12)^	7.6 ± 0.2^(13)^	7.7 ± 0.2^(13)^	7.8 ± 0.2^(12)^	7.8 ± 0.1^(13)^	7.9 ± 0.2^(14)^
BA_INF_ (mg-CaCO_3_/L)	286 ± 78^(13)^	584 ± 20^(11)^	831 ± 59^(13)^	250 ± 47^(17)^	443 ± 59^(15)^	790 ± 82^(13)^
BA_EFF_ (mg-CaCO_3_/L)	902 ± 72^(12)^	1,449 ± 63^(13)^	2050 ± 90^(13)^	837 ± 84^(12)^	1704 ± 70^(13)^	2,287 ± 111^(14)^
TVA_INF_ (mg-HAc/L)	623 ± 111^(13)^	744 ± 63^(11)^	1,307 ± 46^(13)^	640 ± 94^(17)^	1,152 ± 160^(15)^	1,507 ± 106^(13)^
TVA_EFF_ (mg-HAc/L)	39 ± 5^(12)^	52 ± 4^(13)^	99 ± 26^(13)^	39 ± 7^(12)^	68 ± 7^(13)^	91 ± 10^(14)^
HAc (mg/L)	0.0	0.0	71.5	0.0	0.0	63.1
HPr (mg/L)	0.0	0.0	24.2	0.0	0.0	15.3
HBu (mg/L)	0.0	0.0	15.2	0.0	0.0	12.1
OLR_A_ (kg-COD/m^3^/d)	4.1	8.2	12.2	4.1	8.1	12.0
OLR_R_ (kg-COD/m^3^/d)	3.8	7.8	11.7	3.7	7.4	11.2
MPr (mol-CH_4_/m^3^/d)	34.8 ± 4.3^(17)^	101.8 ± 10^(11)^	152.8 ± 15.2^(13)^	22.4 ± 8.0^(8)^	65.9 ± 10.5^(15)^	90.6 ± 10.3^(10)^
Y (mol-CH_4_/kg-COD)	9.2 ± 1.1^(17)^	13.0 ± 2.6^(11)^	13.1 ± 4.0^(13)^	5.6 ± 2.2^(8)^	8.9 ± 2.7^(15)^	8.1 ± 2.6^(10)^
V_CH4_ (mL/cycle)	553 ± 70^(17)^	1,538 ± 155^(11)^	2,348 ± 234^(13)^	338 ± 127^(8)^	1,002 ± 160^(15)^	1,383 ± 168^(10)^
X_CH4_ (%)	88 ± 4^(15)^	86 ± 1.5^(11)^	80 ± 1.5^(13)^	87 ± 4^(8)^	83 ± 1.1^(9)^	83 ± 2^(13)^

Notation: C_INF_, Influent COD concentration; ε_ST_, Total COD removal efficiency; ε_SF_, Filtered COD removal efficiency; C_Carb-INF_, carbohydrate concentration; ε_CT_, total carbohydrate removal efficiency; ε_CF_, filtered carbohydrate removal efficiency; pH_INF_, Influent pH; pH_EFF_, Effluent pH; BA_INF_, influent bicarbonate alkalinity; BA_EFF_, effluent bicarbonate alkalinity; TVA_INF_, influent total volatile acids concentration; TVA_EFF_, effluent total volatile acids concentration; HAc, acetic acid concentration; HPr, Propionic acid concentration; HBu, Butyric acid concentration; OLR_A_, applied organic loading rate; OLR_R_, removed organic loading rate; MPr, Methane molar productivity; Y, molar yield of methane produced per consumed substrate; V_CH4_, methane volume produced per cycle; X_CH4_, Methane composition in biogas. Values in parentheses refer to the number of samples analyzed.

### 2.4 Microbial community analysis

Biomass samples from conditions 3 and 6, the highest organic loading rate (12 kg-COD/m^3^/d) of the mesophilic and thermophilic series, respectively, were collected and stored at −20 °C until analysis. DNA was extracted with the DNeasy PowerSoil Pro Kit (QIAGEN®) according to the manufacturer’s instructions. All samples were amplified with primers targeting both the Bacteria and Archaea domains. Bacterial diversity was assessed with the primer pair 515F/806R (Caporaso et al., 2012), targeting the V4 region of the 16S rRNA gene, under the following thermocycler conditions: 94 °C for 3 min, followed by 30 cycles of 94 °C for 30 s, 50 °C for 30 s, and 72 °C for 30 s, with a final extension at 72 °C for 10 min. The Archaea domain was targeted with the primer pair Ar915aF/Ar1386R (Kittelmann et al., 2013) under the following conditions: 95 °C for 3 min, followed by 25 cycles of 95 °C for 30 s, 62 °C for 30 s, and 72 °C for 30 s, with a final extension at 72 °C for 5 min. All subsequent steps, from purification of the PCR products through Amplicon Sequence Variant (ASV) analysis and taxonomic classification at the genus level, were performed as described by Paulinetti et al. (2023).

Alpha-diversity metrics, including richness (S), the Shannon index (H′, Equation 1), Simpson index (D, Equation 2), and Pielou’s evenness (J′, Equation 3), were calculated to characterize the taxonomic breadth, overall diversity, dominance structure, and distributional evenness of the microbial community under each temperature condition, respectively, based on genus-level relative abundance data, where *S* is the total number of genera and *pᵢ* is the relative abundance of genus *i*.

$$H^{\prime }=-\sum_{i=1}^{S}p_{i}\cdot \ln\left(p_{i}\right)$$ (1)

$$D=\sum_{i=1}^{S}p_{i}^{2}$$ (2)

$$J^{\prime }=\frac{H^{\prime }}{\ln\left(S\right)}$$ (3)

### 2.5 Energy analysis

To identify the reactor configuration that recovered the most energy from soybean molasses, four systems were compared. Two were single-stage methanogenic systems treating raw molasses, operated at mesophilic (S-Meso (Paulinetti et al., 2023)) and thermophilic (S-Thermo (Paulinetti et al., 2024)) temperatures, in which energy was recovered only as methane. The other two were two-stage systems, in which the thermophilic acidogenic reactor (Augusto et al., 2025) was followed by the methanogenic reactor of the present work operated at 30 °C (TS-Thermo/Meso) or 55 °C (TS-Thermo/Thermo); in these, energy was recovered as both hydrogen, in the first stage, and methane, in the second stage. The studies employed the same reactor configuration, analytical methods, operational strategy, and performance indicators. Soybean molasses was provided by the same company across the studies. In addition, the same mesophilic inoculum source was used for the mesophilic reactors, whereas the same thermophilic inoculum source was used for the thermophilic reactors.

For the energy system design, the methanogenic reactor was fed directly with the effluent from the acidogenic reactor. The acidogenic reactor effluent had a concentration of 8,061 ± 401 mg-COD/L (relative standard error = 5.0%), whereas the methanogenic reactor influent had concentrations of 7,781 ± 138 mg-COD/L (relative standard error = 1.8%) and 7,621 ± 401 mg-COD/L (relative standard error = 5.3%) under mesophilic and thermophilic conditions, respectively. Thus, the COD concentrations at the outlet of the acidogenic stage and at the inlet of the methanogenic stage were closely matched, with decreases of 3.5% under mesophilic conditions and 5.5% under thermophilic conditions. These relatively small differences support the consistency of the COD connection between the two stages used in the energy balance comparison.

To ensure a common basis for the energy-yield comparison, the systems were designed based on a soybean molasses mass flow rate of 85 g/d. For the two-stage system, an OLR_A_ of approximately 27 kg-COD/m^3^/d was selected for the acidogenic reactor and approximately 12 kg-COD/m^3^/d for the methanogenic reactor, whereas an OLR_A_ of approximately 14.5 kg-COD/m^3^/d was selected for the single-stage system. Taken together, these similarities provided a consistent basis for comparison between the configurations.

The energy generated by each reactor was obtained from its molar gas productivity and the standard molar enthalpy of combustion (lower heating value) of the corresponding gas, taken as 802.6 kJ/mol for methane (Ramos et al., 2021) and 241.8 kJ/mol for hydrogen (Sementa et al., 2026). The volumetric energy productivity (P_E_, kJ/m^3^/d) of a reactor was calculated with Equation 4:

$$P_{E}=\text{MP}r_{i}\;\cdot {\Delta \text{Hc}}_{i}$$ (4)

where MPr*_i_* is the molar productivity of gas *i* (mol-*i*/m^3^/d) and ΔHc*_i_* its enthalpy of combustion (kJ/mol).

The overall energy yield (γ_E_, kJ/kg-COD), adopted as the main comparison criterion, was defined as the total energy generated by the system divided by the total organic matter removed, according to Equation 5 (Ramos et al., 2021):

$$\gamma_{E}=\frac{\sum {\text{MPr}}_{i}\cdot {\Delta \text{Hc}}_{i}\cdot V_{R\;j}}{\sum {\text{OLR}}_{R\;j}\cdot V_{R\;j}}$$ (5)

where V_R *j*_ is the working volume and OLR_R *j*_ the removed organic loading rate (kg-COD/m^3^/d) of reactor *j*. The summation includes only the methanogenic reactor for the single-stage systems and both the acidogenic and methanogenic reactors for the two-stage systems. To represent the complete treatment process, the two stages were linked on a common organic-matter basis, with the soluble COD leaving the acidogenic reactor taken as the COD entering the methanogenic reactor, and the acidogenic and methanogenic data combined at matching organic loading rates.

## 3 Results

An overview of the methanogenic reactor performance across the evaluated conditions is presented in Table 2, which summarizes the key operational stability and methane production parameters discussed throughout this section.

### 3.1 Second-stage methanogenic reactor

In both the mesophilic (Conditions 1–3) and thermophilic (Conditions 4–6) series, increasing the applied organic loading rate (OLR_A_) from 4 to 12 kg-COD/m^3^/d slightly increased the COD removal efficiency (Table 2), which rose from 91% to 95% (total) under mesophilic conditions and from 87% to 91% under thermophilic conditions. Carbohydrate removal was essentially complete and insensitive to OLR_A_ in both series, remaining between 97% and 99%, consistent with the readily fermentable nature of the residual sugars in the acidogenic effluent.

The process remained stable across the entire OLR_A_ range, with no accumulation of volatile acids. In all conditions, the effluent total volatile acids concentration (TVA_EFF_, 39–99 mg-HAc/L) was markedly lower than that of the influent (TVA_INF_, 623–1,507 mg-HAc/L), indicating effective consumption of the acids carried over from the acidogenic stage during methanogenesis. Accordingly, individual acids were below detection up to 8 kg-COD/m^3^/d in both series and appeared only at the highest load (12 kg-COD/m^3^/d), and even then at low concentrations (acetic acid of 71.5 and 63.1 mg/L for the mesophilic and thermophilic series, respectively). Effluent pH remained within the optimal methanogenic range (7.5–7.9), and bicarbonate alkalinity was generated within the reactor (BA_EFF_ > BA_INF_ in every condition), confirming the buffering capacity of the second stage.

The methane content of the biogas was high in all conditions but decreased slightly as the OLR_A_ increased, from 88% to 80% under mesophilic operation and from 87% to 83% under thermophilic operation, a trend consistent with the greater CO_2_ release that accompanies the more intense acidogenic and methanogenic activity at higher organic loads.

Under mesophilic conditions, raising the OLR_A_ increased both methane productivity and yield. Productivity rose more than fourfold, from 34.8 to 152.8 mol-CH_4_/m^3^/d as the OLR_A_ increased from 4 to 12 kg-COD/m^3^/d, while the methane yield increased from 9.2 to 13.1 mol-CH_4_/kg-COD. This concurrent gain in productivity and yield indicates that the additional organic matter was efficiently channeled toward methane, since a higher OLR_A_ increases substrate availability and stimulates the metabolic activity of the methanogenic consortium (Bezerra et al., 2009).

Under thermophilic conditions, methane productivity also increased with the OLR_A_, reaching 90.6 mol-CH_4_/m^3^/d at 12 kg-COD/m^3^/d. The methane yield increased from 5.6 to 8.9 mol-CH_4_/kg-COD as the OLR_A_ rose from 4 to 8 kg-COD/m^3^/d (Conditions 4 and 5); however, a further increase to 12 kg-COD/m^3^/d (Condition 6) caused the yield to decline to 8.1 mol-CH_4_/kg-COD. This decrease suggests that, at the highest thermophilic load, part of the organic matter was only partially converted, being diverted toward volatile acids and cellular biomass rather than being completely converted to methane, as reflected by the higher TVA_EFF_ in Condition 6 (91 mg-HAc/L) than in Condition 5 (68 mg-HAc/L). The contrast with the mesophilic series, in which yield increased monotonically with load, indicates a more efficient and robust conversion of organic matter at 30 °C.

Overall, the mesophilic reactor outperformed the thermophilic one in both methane productivity and yield across the whole OLR_A_ range. At 12 kg-COD/m^3^/d, mesophilic productivity was 68.7% higher (152.8 versus 90.6 mol-CH_4_/m^3^/d) and the corresponding yield 61.7% higher (13.1 versus 8.1 mol-CH_4_/kg-COD) than under thermophilic operation. This superior performance is consistent with the greater microbial diversity typically sustained in mesophilic systems, which supports more robust metabolic networks through synergistic interactions among functional groups. Thermophilic conditions, by contrast, select more specialized populations with lower metabolic redundancy, rendering the system more vulnerable to stressors such as organic overload or volatile-acid accumulation; although thermophilic communities can be efficient once fully adapted, their operational stability is generally lower than that of the more resilient and flexible mesophilic systems (Batista et al., 2023).

### 3.2 Microbial community diversity

The microbial community was characterized at the highest organic loading rate (12 kg-COD/m^3^/d) of each series, allowing a direct comparison between the mesophilic (Condition 3, 30 °C) and thermophilic (Condition 6, 55 °C) reactors under an otherwise identical operating condition. Temperature was the primary factor shaping microbial community composition: the mesophilic and thermophilic reactors exhibited a profound restructuring of the bacterial community and a pronounced shift in the methanogenic pathway between 30 °C and 55 °C (Figure 2). For clarity, Figure 2 displays only genera with a relative abundance greater than 0.1%. Richness and diversity indices were calculated using the complete genus-level abundance data and are presented in Table 3 for both microbial domains.

**FIGURE 2:**
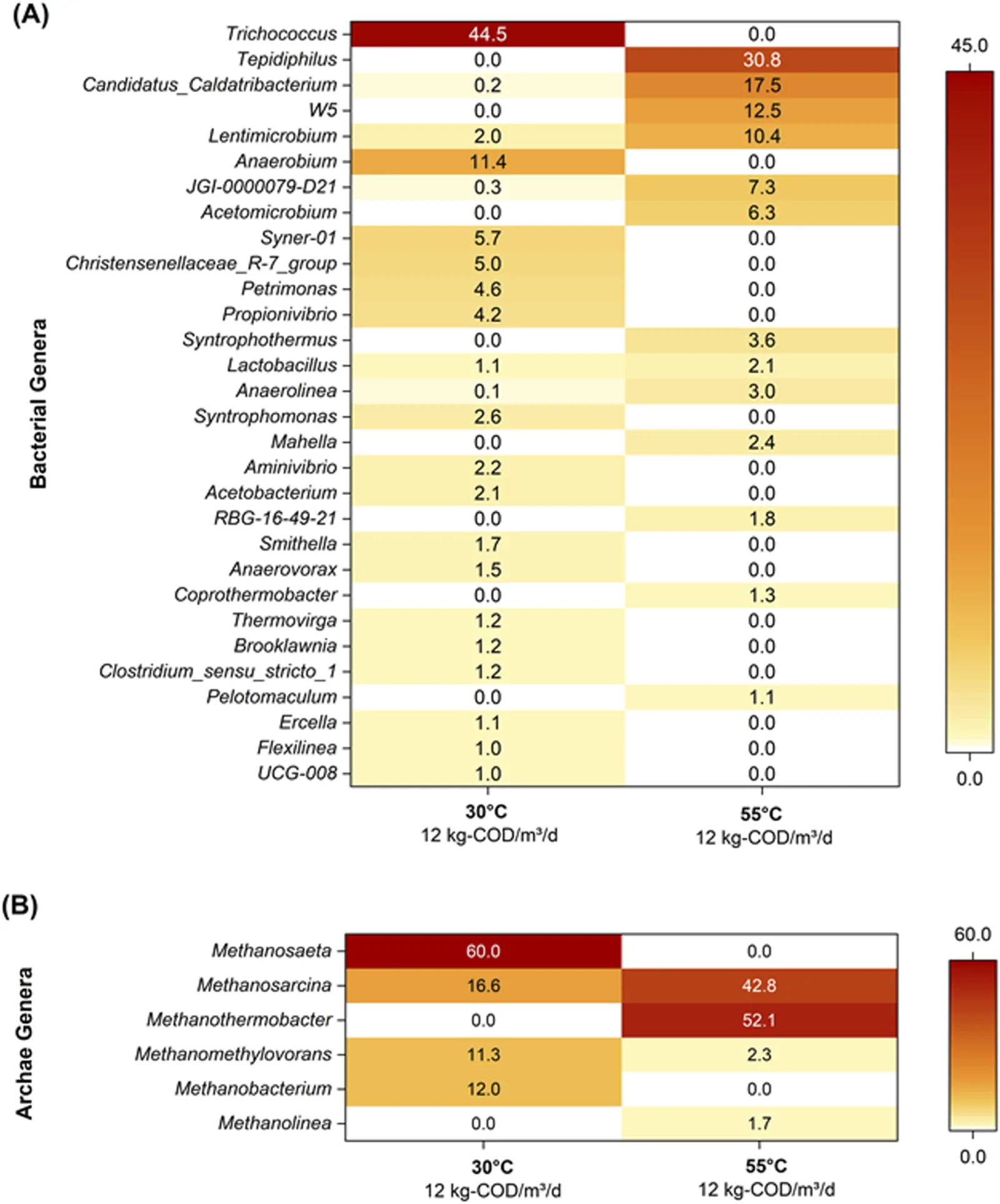
Bacterial **(A)** and Archaea **(B)** community composition at genus level.

**TABLE 3:** Alpha-diversity indices of the bacterial and archaeal communities under mesophilic and thermophilic conditions at 12 kg-COD/m^3^/d.

Domain	Condition	Richness (S)	Shannon (H′)	Simpson (D)	Pielou (J′)
Bacteria	Mesophilic	30	2.26	0.225	0.666
	Thermophilic	13	2.10	0.165	0.819
Archaea	Mesophilic	4	1.11	0.415	0.797
	Thermophilic	4	0.86	0.455	0.620

Overall, the mesophilic reactor exhibited greater bacterial and archaeal diversity than the thermophilic reactor, as indicated by bacterial richness (30 vs. 13 genera), bacterial Shannon diversity (H′ = 2.26 vs. 2.10), and archaeal Shannon diversity (H′ = 1.11 vs. 0.86). However, the two microbial domains responded differently to temperature-driven selection. In the bacterial community, the thermophilic condition was associated with a reduction of more than 50% in genus-level richness (13 vs. 30 genera), whereas the remaining genera showed greater evenness (Pielou’s J′ = 0.819 vs. 0.666) and lower dominance (Simpson’s D = 0.165 vs. 0.225). Thus, although the thermophilic bacterial community comprised substantially fewer genera, the relative abundances were more evenly distributed among the remaining genera. The higher bacterial dominance in the mesophilic reactor was associated with the greater relative abundance of *Trichococcus* (44.5%), whereas *Tepidiphilus* was the most abundant genus in the thermophilic reactor but accounted for a smaller proportion of the community (30.8%), resulting in a relatively greater contribution of the remaining genera to the total bacterial abundance. In contrast, archaeal richness remained unchanged between the two reactors (S = 4 genera), whereas the thermophilic community exhibited lower evenness (Pielou’s J′ = 0.620 vs. 0.797) and higher dominance (Simpson’s D = 0.455 vs. 0.415). This pattern reflects differences in the relative abundance structure of the four archaeal genera, with greater dominance of *Methanothermobacter* and *Methanosarcina* under the thermophilic condition (Figure 2B). Collectively, these results demonstrate that the bacterial and archaeal communities exhibited distinct responses to temperature-driven selection: the thermophilic condition was associated with lower bacterial richness but greater evenness and lower dominance, whereas the archaeal community maintained the same richness but exhibited lower evenness and greater dominance.

In the mesophilic reactor (Figure 2A), the bacterial community was dominated by *Trichococcus* (44.5%), an acidogenic genus capable of converting substrates into acetate, formate, and ethanol during the early steps of anaerobic digestion (Zhang et al., 2022). Its dominance has been previously reported in anaerobic systems treating dairy wastewater (Zieliński et al., 2021), reinforcing its ecological relevance in mesophilic sludge communities. The bacterial community was further complemented by *Anaerobium* (11.4%), *Petrimonas* (4.6%), and *Propionivibrio* (4.2%), all strictly anaerobic mesophilic fermenters with an established role in anaerobic digestion reactors. *Anaerobium* is a typical hydrolytic fermentation bacterium in anaerobic bioreactors (Wang et al., 2023). *Petrimonas* is a mesophilic anaerobic fermenter (Grabowski et al., 2005) previously identified as a dominant genus in mesophilic anaerobic digesters treating food waste (Kim et al., 2018). *Propionivibrio*, in turn, displays a fermentative metabolism with acetate and propionate as end products (Albertsen et al., 2016), contributing to the substrate flux toward acetogenesis. Together, these genera constitute a functionally diverse and well-structured bacterial community, characteristic of stable mesophilic anaerobic digestion systems (Kim et al., 2018).

In the thermophilic reactor (Figure 2A), the bacterial community was dominated by *Tepidiphilus* (30.8%), a strictly thermophilic genus whose prevalence at 55 °C has been documented in thermophilic anaerobic reactors, where it constituted over 28% of the bacterial community (Paulinetti et al., 2024), and has also been reported among the dominant genera in thermophilic anaerobic digestion of *Arundo donax* (Lei et al., 2020). The community was further complemented by *Candidatus Caldatribacterium* (17.5%), W5 (12.5%), and *Lentimicrobium* (10.4%). *Candidatus Caldatribacterium* is an anaerobic fermentative lineage previously identified among dominant acidogenic genera in thermophilic anaerobic digestion systems (Ao et al., 2019; Mikheeva et al., 2024). *Lentimicrobium*, in turn, ferments small organic compounds such as glucose to produce volatile fatty acids (Ni et al., 2021), and its enrichment under thermophilic conditions has been previously reported (Kozera et al., 2026).

Overall, the mesophilic reactor harbored a more diverse and functionally redundant bacterial community, with abundances distributed across numerous hydrolytic and fermentative genera (Wu et al., 2020; Ao et al., 2021). In contrast, the thermophilic reactor was characterized by a more restricted community structure, dominated by a few thermophilic specialists, a pattern attributed to the stronger selective pressure exerted by elevated temperature, which reduces microbial richness and diversity while favoring the proliferation of thermoadapted genera (Li et al., 2025; Liu et al., 2025).

These community differences are consistent with the performance data reported in Section 3.1. In the mesophilic reactor, the functionally diverse bacterial consortium sustained higher methane productivity (152.8 mol-CH_4_/m^3^/d) and yield (13.1 mol-CH_4_/kg-COD) at 12 kg- COD/m^3^/d, with organic matter being efficiently converted into methane and low volatile acid accumulation in the effluent (99 mg-HAc/L). In the thermophilic reactor, despite reaching a methane productivity of 90.6 mol-CH_4_/m^3^/d, the methane yield declined to 8.1 mol-CH_4_/kg-COD at the highest organic loading rate, suggesting that part of the organic matter was diverted toward volatile fatty acid accumulation and biomass synthesis rather than being fully converted into methane (André et al., 2026). This pattern is reinforced by the predominance of *Tepidiphilus*, a genus capable of accumulating poly-β-hydroxybutyrate (PHB) as an intracellular reserve under restricted growth conditions (Manaia et al., 2003; Paulinetti et al., 2024), suggesting that part of the carbon may have been diverted toward cellular storage rather than being channeled into methanogenesis. Although PHB content was not measured in the present study, this potential carbon allocation is consistent with the more specialized and less redundant thermophilic community structure.

The archaeal communities reflected two distinct methanogenic strategies (Figure 2B), as expected in anaerobic digestion systems, where archaea are primarily responsible for methane production (Batista et al., 2023).

Genus-level classification revealed distinct profiles between the two reactors. In the mesophilic reactor (30 °C), the community was dominated by the strictly acetoclastic *Methanosaeta* (60.0%), whose predominance is favored in mesophilic digesters operating under low acetate concentrations (Conklin et al., 2006), complemented by the hydrogenotrophic *Methanobacterium* (12.0%) (Amin et al., 2021), the methylotrophic *Methanomethylovorans* (11.3%) (Lomans et al., 1999), and the metabolically versatile *Methanosarcina* (16.6%) (Zhang et al., 2019). This composition indicates that methane was produced predominantly through the direct conversion of acetate into methane (acetoclastic pathway), supported by parallel hydrogenotrophic and methylotrophic routes.

In the thermophilic reactor (55 °C), *Methanosaeta* was absent and the community was dominated by the strictly hydrogenotrophic archaeon *Methanothermobacter* (52.1%), reported as the predominant archaeal genus in thermophilic anaerobic digesters, producing methane from the consumption of CO_2_ and H_2_ (Wang et al., 2024). *Methanosarcina* (42.8%) was also present in high abundance, reflecting its metabolic versatility: this genus is capable of using H_2_ and CO_2_, methylated compounds, or acetate for methanogenesis (Welte and Deppenmeier, 2011), which allows it to proliferate under varying substrate conditions. The disappearance of *Methanosaeta* and the dominance of hydrogenotrophic methanogenesis indicate that, at 55 °C, acetate was largely consumed through syntrophic acetate oxidation (SAO) coupled to hydrogenotrophic archaea, rather than through direct acetoclastic conversion. This mechanism is supported by the presence of *Coprothermobacter* in the thermophilic bacterial community, a genus that generates hydrogen through acetate oxidation and establishes syntrophic interactions with hydrogenotrophic methanogens such as *Methanothermobacter thermautotrophicus* (Deligiannis et al., 2024).

The microbial patterns observed under thermophilic conditions are consistent with recent findings from other anaerobic digestion systems. Nakamura et al. (2026) reported a syntrophic association between *Coprothermobacter* and the hydrogenotrophic methanogen *Methanobacterium* during thermophilic operation, suggesting that this type of metabolic interaction may also occur with substrates other than soybean molasses. This finding is consistent with the SAO–*Methanothermobacter* association observed at 55 °C in the present study and supports the occurrence of hydrogenotrophic methanogenesis under thermophilic conditions. In addition to differences in microbial communities between stable temperature conditions, recent studies have also examined how changes in operating temperature affect methanogenic systems (Sudiartha et al., 2023). Sudiartha et al. (2023) reported that downward temperature shifts reduced methane production by 16%–33%, while upward shifts led to a decrease of up to 83%, demonstrating that the response of methanogenic systems depends on the direction of the shift and the initial operating temperature. Although the present study evaluated fixed-temperature operation rather than dynamic temperature conditions, future studies could investigate such perturbations to assess the robustness of the system.

This distinction is functionally relevant to system performance. In the mesophilic reactor, the dominance of acetoclastic methanogenesis by *Methanosaeta*–a high-affinity acetate scavenger capable of operating efficiently at low acetate concentrations (Amin et al., 2021) – sustained a more efficient conversion of organic matter into methane. In the thermophilic reactor, methane formation depended on the SAO–hydrogenotrophic route (McDaniel et al., 2023), a two-step pathway favored under low hydrogen partial pressure (Hao et al., 2013) and generally described as thermodynamically tighter than direct acetoclastic cleavage (Diniz et al., 2026), a constraint that may have contributed to the lower methane yield observed at the highest organic loading rate.

### 3.3 Energy analysis

To determine which reactor configuration recovered the most energy from soybean molasses, the overall energy yield (γ_E_) of four systems was compared: the two single-stage methanogenic systems treating raw molasses, operated at 30 °C (Paulinetti et al., 2023) and 55 °C (Paulinetti et al., 2024); and the two two-stage systems, in which the thermophilic acidogenic reactor (Augusto et al., 2025) was coupled to the methanogenic reactor of the present work operated at 30 °C or 55 °C. For the two-stage systems, γ_E_ accounts for the energy recovered as both hydrogen, in the first stage, and methane, in the second, referred to the total organic matter removed throughout the complete two-stage process (Figure 3).

**FIGURE 3:**
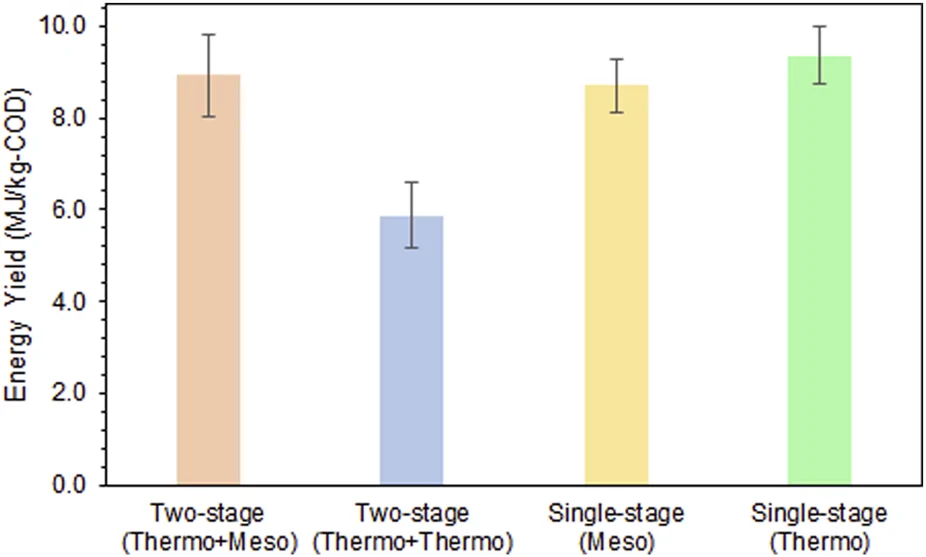
Energy yield of the four anaerobic digestion systems.

The single-stage thermophilic system achieved the highest energy yield (9.36 ± 0.63 MJ/kg-COD), followed by the two-stage thermophilic–mesophilic system (8.94 ± 0.89 MJ/kg-COD) and the single-stage mesophilic system (8.71 ± 0.58 MJ/kg-COD). No clear difference was observed between three configurations based on the stable-phase cycle values (one-way ANOVA, p > 0.05), indicating that, on an energy-per-COD-removed basis, coupling a thermophilic acidogenic stage to a mesophilic methanogenic stage recovered essentially as much energy as either single-stage system alone. The two-stage thermophilic–thermophilic system was clearly inferior (5.86 ± 0.71 MJ/kg-COD), reflecting the poorer methane productivity and yield of the thermophilic second stage at high load (Section 3.1).

This poorer methane productivity and yield of the thermophilic second stage, however, does not extend to the single-stage thermophilic system, which achieved a high energy yield (9.36 ± 0.63 MJ/kg-COD) despite relying on a similarly hydrogenotrophic-dominated archaeal community (Paulinetti et al., 2024). The distinction lies in the interaction between this metabolic dependence on hydrogen and the substrate available to each reactor. The thermophilic second-stage reactor of the present study received effluent already fermented by the upstream thermophilic acidogenic reactor (Augusto et al., 2025), consisting predominantly of volatile acids with a reduced potential to generate additional hydrogen through oxidation compared to the original substrate. This limited hydrogen-generating potential compounded the intrinsic thermodynamic constraints of the SAO–hydrogenotrophic pathway (Section 3.2), thereby restricting methane formation and, consequently, energy recovery in the thermophilic–thermophilic configuration. In contrast, the single-stage thermophilic reactor was fed directly with soybean molasses, retaining the full hydrogen-generating potential of the original substrate to sustain methanogenesis. This comparison indicates that the lower methane production performance of the thermophilic second-stage reactor arises not from the hydrogenotrophic pathway alone, but from its combination with the reduced hydrogen-generating potential of the substrate resulting from upstream acidogenic fermentation.

The central finding is therefore that splitting the process into two stages did not increase the overall energy yield relative to single-stage methanogenesis. This outcome is consistent with the report of Lovato et al. (2020), who, co-digesting whey and glycerin in AnSBBRs, found that a two-stage hydrogen-plus-methane system yielded no more energy per removed COD than a single-stage methane system, and concluded that phase separation is energetically advantageous only when the single-stage system performs poorly, specifically, when its methane yield falls below about 56% of the theoretical maximum. For soybean molasses, both single-stage systems converted organic matter to methane efficiently, leaving limited potential for a two-stage system to improve the energy balance. From an energy recovery perspective, an important explanation is thermodynamic: the lower heating value of hydrogen (241.8 kJ/mol) (Sementa et al., 2026) is roughly one-third that of methane (802.6 kJ/mol) (Ramos et al., 2021), so converting part of the substrate into hydrogen during the first stage limits the energetic contribution of the acidogenic reactor, and the additional methane recovered downstream only compensates for, rather than exceeds, the energy that single-stage methanogenesis would have obtained directly. This energetic disparity remains evident on a practical volumetric basis. Methane’s higher heating value (37.74 MJ/Nm^3^) is approximately three times that of hydrogen (12.75 MJ/Nm^3^) (Nigro et al., 1997), underscoring methane’s inherent advantage as an energy carrier. Hydrogen’s comparatively low volumetric energy content also contributes to the well-recognized challenges associated with its storage and transport relative to methane.

Within the two-stage systems, however, the temperature pairing was decisive. The thermophilic–mesophilic configuration recovered 53% more energy than the thermophilic–thermophilic one (8.94 versus 5.86 MJ/kg-COD) and matched both single-stage systems. This superiority was driven entirely by the second stage: the mesophilic methanogenic reactor outperformed the thermophilic reactor in methane conversion efficiency and process stability (Section 3.1), consistent with the greater functional redundancy and shock resistance of mesophilic methanogenic communities (Section 3.2). The same pairing was identified as optimal by Ramos et al. (2021) for the two-stage digestion of sugarcane vinasse, where a thermophilic acidogenic reactor followed by a mesophilic methanogenic reactor gave the highest energy yield under high-load conditions. This configuration is also favorable in practice, since soybean molasses leaves the soy protein concentrate process at approximately 50 °C (close to the operating temperature of the thermophilic acidogenic stage) while a mesophilic second stage helps cool the digestate toward the ambient temperature required for downstream handling.

## 4 Discussion

The anaerobic digestion of soybean molasses in a second-stage methanogenic AnSBBR was stable and efficient across the entire range of applied organic loading rates, in both the mesophilic and thermophilic series. The acidified effluent of the thermophilic acidogenic reactor, rich in volatile acids and ethanol, was readily converted to methane: total volatile acids were consumed almost completely (effluent concentrations of 39–99 mg-HAc/L against influent values of 623–1,507 mg-HAc/L), bicarbonate alkalinity was generated within the reactor, and the effluent pH remained within the optimal methanogenic range. This behavior confirms one of the principal advantages claimed for two-stage systems: by absorbing the acid load and pH fluctuations of the acidified influent, the methanogenic stage operated under buffered, near-neutral conditions even at the highest loads (Lovato et al., 2020; Cremonez et al., 2021).

Temperature, rather than organic load, was the decisive operational variable. Under mesophilic conditions, both methane productivity and yield rose with loading, reaching 152.8 mol-CH_4_/m^3^/d and 13.1 mol-CH_4_/kg-COD at 12 kg-COD/m^3^/d, indicating that the additional organic matter was efficiently channeled to methane. Under thermophilic conditions, productivity also increased with load but the yield declined at the highest loading, consistent with a diversion of carbon toward volatile acids and cellular biomass rather than complete conversion to methane. As a result, the mesophilic reactor outperformed the thermophilic one in both productivity (by 69%) and yield (by 62%) at 12 kg-COD/m^3^/d. This contrast reproduces, at the methanogenic-stage level, the performance-versus-stability trade-off previously reported for the single-stage digestion of this same substrate (Paulinetti et al., 2024) and for other agro-industrial wastewaters (Ramos et al., 2021; Batista et al., 2023): thermophilic operation offers no advantage, and a clear disadvantage at high load, when the methanogenic community is not fully buffered against overload.

The methane productivity achieved in the present study (152.8 mol-CH_4_/m^3^/d) falls within the range reported for high-rate anaerobic systems treating readily biodegradable agro-industrial wastewaters, particularly those operated in AnSBBR and related configurations. Paulinetti et al. (2024), investigating single-stage anaerobic digestion of soybean molasses under similar operational conditions, reported maximum methane productivities of 140.9 mol-CH_4_/m^3^/d for mesophilic operation and 144.6 mol-CH_4_/m^3^/d for thermophilic operation at comparable or even higher organic loading rates (15 kg-COD/m^3^/d). In comparison, the present two-stage configuration achieved a similar or slightly higher volumetric performance at a lower applied organic load, indicating efficient conversion of the acidified effluent in the methanogenic stage.

Similarly, Lovato et al. (2020) reported methane productivities of 185.9 mol-CH_4_/m^3^/d in an AnSBBR treating the acidogenic effluent from whey-glycerol co-digestion under fed-batch operation and comparable loading conditions. Although slightly higher than the values obtained in the present study, their results are of the same order of magnitude and confirm that high methane production rates can be consistently achieved in AnSBBR systems fed with acidified intermediates from highly biodegradable substrates.

In this context, the performance observed in the present study confirms that soy molasses-derived effluents are highly suitable for methanogenic conversion in two-stage systems and that the proposed configuration maintains competitive methane production rates when compared with both single-stage and alternative two-stage processes reported in the literature.

The microbial analysis explained this behavior and, when set alongside the communities reported for the two single-stage systems treating the same molasses (Paulinetti et al., 2023; 2024), revealed a consistent pattern across all four configurations: community structure was governed primarily by temperature, and only secondarily by feed composition or the number of process stages. In both mesophilic reactors (the single-stage system fed raw molasses and the two-stage second stage fed acidified effluent) the bacterial community was led by the metabolically versatile hydrolytic–acidogenic genus *Trichococcus* and supported by a diverse assemblage of fermentative and syntrophic acid-handling bacteria, while the archaeal community was dominated by the strictly acetoclastic *Methanosaeta*. Methane production in this mesophilic reactor was therefore mainly driven by the direct cleavage of acetate, a single enzymatic step that operates efficiently even at the low acetate concentrations maintained in a well-buffered reactor. In contrast, both thermophilic reactors converged toward a narrower bacterial assemblage dominated by *Tepidiphilus*, accompanied by the thermophilic fermenters (*Candidatus Caldatribacterium, Acetomicrobium*) and the proteolytic, acetate-oxidizing *Coprothermobacter*, while the archaeal community shifted to the strictly hydrogenotrophic *Methanothermobacter* and the metabolically versatile *Methanosarcina*. This community composition in the thermophilic reactor is consistent with acetate being consumed predominantly through syntrophic acetate oxidation (SAO) coupled with hydrogenotrophic methanogenesis rather than by direct acetoclastic cleavage. Unlike the acetoclastic pathway, this route involves two sequential and thermodynamically coupled reactions, acetate oxidation followed by hydrogenotrophic methanogenesis, which remain favorable only under very low hydrogen partial pressure (Hao et al., 2013). Consequently, the thermophilic pathway is inherently more susceptible to transient organic or hydraulic overload, favoring volatile fatty acid accumulation at the highest loading rate. The predominance of *Tepidiphilus*, a genus reported to accumulate carbon as poly-β-hydroxybutyrate under growth-limiting conditions, may be associated with the allocation of part of the organic matter toward intracellular reserves, potentially contributing to the lower methane yield observed at high thermophilic organic loading rates. Although targeted microbial enrichment strategies may enhance thermophilic methane production by increasing the abundance or activity of key functional populations, such as syntrophic microorganisms that support hydrogenotrophic methanogenesis, their effectiveness may be limited by the intrinsic thermodynamic constraints of this syntrophic pathway.

The energy analysis (Section 3.3) showed that splitting the process into two stages did not increase the overall energy yield relative to single stage methanogenesis, with no clear difference observed among the single-stage systems and the thermophilic–mesophilic configuration, while the thermophilic–thermophilic system was markedly inferior. Because both single-stage systems already converted molasses to methane efficiently, there was little margin for a two-stage system to improve the energy balance; moreover, the low heating value of hydrogen limits the energy that the acidogenic stage can contribute, so the methane recovered downstream only compensates for, rather than exceeds, the energy that single-stage methanogenesis obtains directly. This conclusion is consistent with the findings of Lovato et al. (2020), who reported that, for whey and glycerin, two-stage anaerobic digestion improves energy yield only when the corresponding single-stage process performs poorly. Within the two-stage systems, however, the temperature pairing was decisive: the thermophilic–mesophilic configuration recovered 53% more energy than the thermophilic–thermophilic one and matched both single-stage systems, the same pairing identified as optimal for sugarcane vinasse by Ramos et al. (2021). This outcome reflects a clear division of roles between the two stages. Thermophilic operation offered no performance advantage at the methanogenic stage under any evaluated condition, whereas mesophilic operation consistently sustained the most robust and efficient methane recovery, whether applied in a single stage or as the second stage of a two-stage system, owing to the diverse, acetoclastic-dominated community it supports. Thermophilic operation remains advantageous only at the acidogenic stage, where it aligns with the temperature at which molasses leaves the soy protein concentrate production process (approximately 50 °C), while the mesophilic methanogenic stage progressively cools the digestate toward the ambient temperature required for downstream handling or discharge. Since single stage and two stage systems showed no clear difference in energy recovery, their selection should be guided by factors other than this criterion. The thermophilic acidogenic followed by mesophilic methanogenic configuration enables the recovery of a separate hydrogen stream, a clean energy carrier with broader industrial applications than methane. In addition, the two-stage configuration enhances process stability through improved buffering capacity. In contrast, single stage mesophilic or thermophilic methanogenesis offers the simplest reactor design and process operation.

## Data Availability

The original contributions presented in the study are included in the article, further inquiries can be directed to the corresponding author.
